# Unsupervised machine learning can delineate central sulcus by using the spatiotemporal characteristic of somatosensory evoked potentials

**DOI:** 10.1088/1741-2552/abf68a

**Published:** 2021-04-29

**Authors:** Priscella Asman, Sujit Prabhu, Dhiego Bastos, Sudhakar Tummala, Shreyas Bhavsar, Thomas Michael McHugh, Nuri Firat Ince

**Affiliations:** 1 Department of Biomedical Engineering, University of Houston, Houston, TX, United States of America; 2 Department of Neurosurgery, UT MD Anderson Cancer Center, Houston, TX, United States of America; 3 Department of Anesthesiology, UT MD Anderson Cancer Center, Houston, TX, United States of America

**Keywords:** functional mapping, machine learning, somatosensory evoked potentials

## Abstract

**Objective.:**

Somatosensory evoked potentials (SSEPs) recorded with electrocorticography (ECoG) for central sulcus (CS) identification is a widely accepted procedure in routine intraoperative neurophysiological monitoring. Clinical practices test the short-latency SSEPs for the phase reversal over strip electrodes. However, assessments based on waveform morphology are susceptible to variations in interpretations due to the hand area’s localized nature and usually require multiple electrode placements or electrode relocation. We investigated the feasibility of unsupervised delineation of the CS by using the spatiotemporal patterns of the SSEP captured with the ECoG grid.

**Approach.:**

Intraoperatively, SSEPs were recorded from eight patients using ECoG grids placed over the sensorimotor cortex. Neurosurgeons blinded to the electrophysiology identified the sensory and motor gyri using neuronavigation based on sulcal anatomy. We quantified the most discriminatory time points in SSEPs temporal profile between the primary motor (M1) and somatosensory (S1) cortex using the Fisher discrimination criterion. We visualized the amplitude gradient of the SSEP over a 2D heat map to provide visual feedback for the delineation of the CS based on electrophysiology. Subsequently, we employed spectral clustering using the entire the SSEP waveform without selecting any time points and grouped ECoG channels in an unsupervised fashion.

**Main results.:**

Consistently in all patients, two different time points provided almost equal discrimination between anterior and posterior channels, which vividly outlined the CS when we viewed the SSEP amplitude distribution as a spatial 2D heat map. The first discriminative time point was in proximity to the conventionally favored ~20 ms peak (N20), and the second time point was slightly later than the markedly high ~30 ms peak (P30). Still, the location of these time points varied noticeably across subjects. Unsupervised clustering approach separated the anterior and posterior channels with an accuracy of 96.3% based on the time derivative of the SSEP trace without the need for a subject-specific time point selection. In contrast, the raw trace resulted in an accuracy of 88.0%.

**Significance.:**

We show that the unsupervised clustering of the SSEP trace assessed with subdural electrode grids can delineate the CS automatically with high precision, and the constructed heat maps can localize the motor cortex. We anticipate that the spatiotemporal patterns of SSEP fused with machine learning can serve as a useful tool to assist in surgical planning.

## 1. Introduction

Judicious cortical mapping of an entire craniotomy is especially important in the surgical planning of patients with gliomas that are located within or adjacent to the Rolandic cortex [1–4]. This is done in order to minimize the risk of transient or permanent neurologic compromise that might negatively affect one’s exteroceptive perception or lead to post-operative functional deficit [5–9]. Intraoperative cortical mapping is widely used in clinical practice for central sulcus (CS) localization via the gold standard median nerve somatosensory evoked potentials phase reversal technique (MSSEP-PRT) [10–17]. The technique is based on the reversal in polarity of the median thalamocortical somatosensory evoked potentials (SSEPs) at around 20 ms (termed as N20), at the boundaries of the CS [18–22]. The somatosensory evoked potential phase reversal (SSEP-PR) consists of the cortical N20 potential when recorded from the postcentral gyrus, and the cortical P20 when recorded from the precentral gyrus [23, 24].

The routine clinical assessment of the phase reversal uses electrocorticography (ECoG) strip electrodes (such as the 1 × 8 and 1 × 4 array with 5–10 mm electrode spacing) [4, 24–28]. However, common factors such as anatomical variability of the hand area position, the complexity of the exposed brain during surgery, and the potential distortion of normal anatomy caused by the tumor or tumor-related edema can result in an inability to position the electrode array precisely over the hand area. This can lead to questionable or misinterpreted SSEP-PR [24, 29, 30]. The investigator relies on a rapid intraoperative interpretation of the results, as several recordings in different locations are needed to find the appropriate position to target the somatosensory cortex [25, 29–33]. This increases the risks of hemorrhage or trauma [28, 34]. Moreover, the routine clinical MSSEP-PRT relies only on the amplitude construal of the conventional N20 (posterior) and P20 (anterior) between 2 adjacent contacts to delineate the CS [4, 24, 35]. When there is unsolvable doubt, direct cortical stimulation (DCS) is used to confirm the delineation success [36–38]. Although DCS ensures a thorough investigation of cortical function, it comes with a risk of potentially triggering seizure activities that could jeopardize the surgical procedure or complicate the motor monitorization [39, 40].

This study aims to express the spatiotemporal patterns of SSEPs over ECoG grids with heat maps based on the hypothesis that the heat maps viewed over a high channel grid provide a non-ambiguous view of the CS. As a first step, we quantified the most discriminative time points in the SSEP trace that can distinguish between channels located anterior (M1) and posterior (S1) to the CS. Later, to overcome the constraints of peak and latency interpretations in the high channel ECoG grid recordings, we employed an unsupervised machine learning technique based on spectral clustering to group channels in an automated fashion. We investigated the viability of delineating the CS with the spatiotemporal patterns of the SSEP trace instead of the conventional technique of manual peak and latency tracking to facilitate the discrimination between sensory and motor areas during presurgical evaluation. We noted that while 2D SSEP heat maps provide a clear view of the CS at subject-specific time points, the unsupervised machine learning approach could group the channels located in M1 and S1 in an automated fashion without selecting the subject’s definite time points in the SSEP trace.

## 2. Materials and methods

The study protocol was reviewed and approved by the Institutional Review Boards (IRB) of The University of Texas MD Anderson Cancer Center and The University of Houston. Patients consented to functional mapping before surgery and were informed of the characteristic thumb twitching, they would experience from the median nerve stimulation when they are awoken intraoperatively.

### 2.1. Inclusion and exclusion criteria

The inclusion criteria of the present study consisted of patients with primary brain tumors within or adjacent to the peri Rolandic area (a) undergoing craniotomy in the vicinity of the sensorimotor area for tumor resection (b) with age ranging from 20 to 70 years (c) use of high density ECoG recording during the sedated or awake state, (d) real-time functional cortical mapping for the primary somatosensory hand area by measurement of the median nerve SSEPs.

### 2.2. Surgical procedure

**Figure 1.**
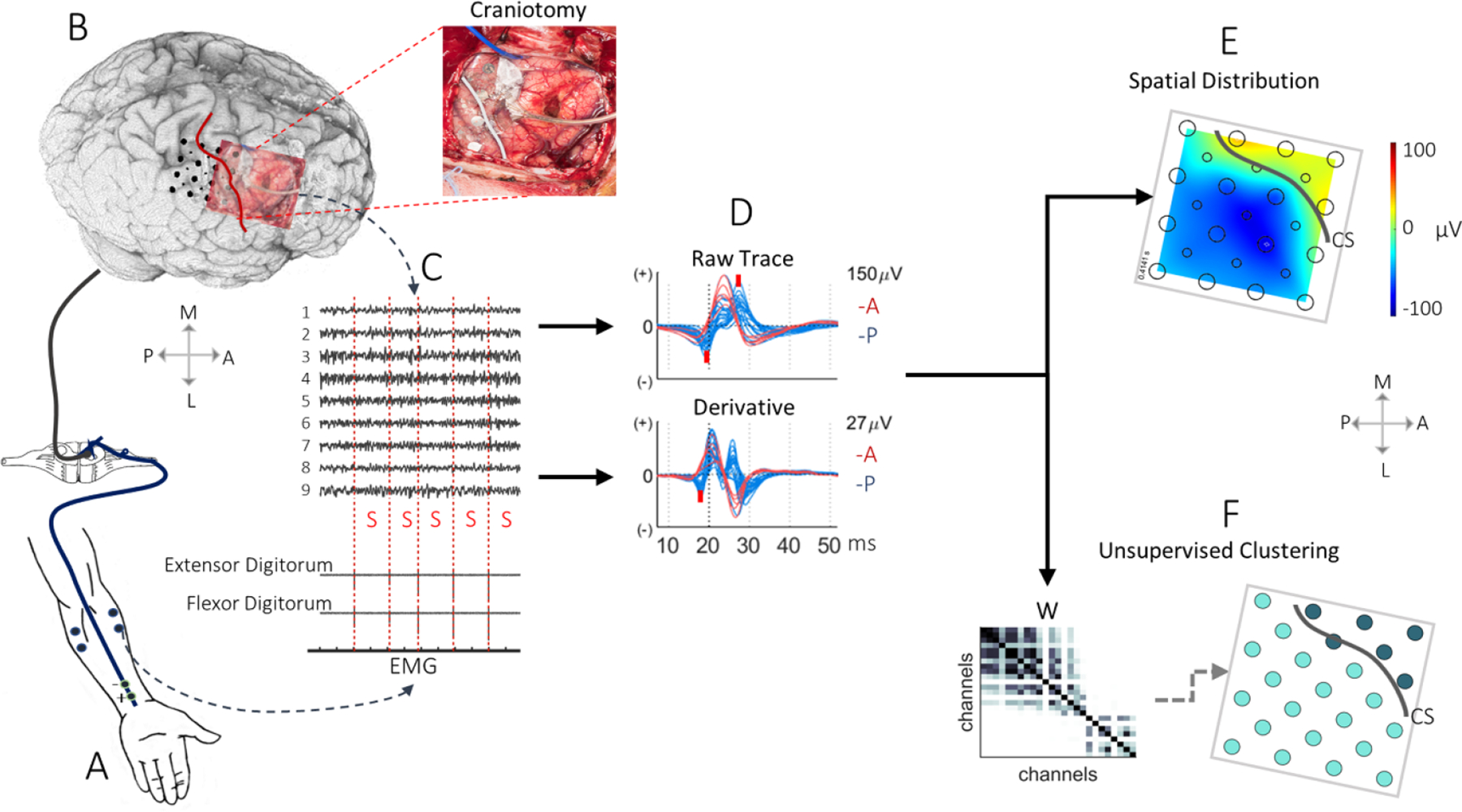
Pipeline of analysis on the central sulcus (CS) localization via MSSEP-PRT. (A) The electrical stimulus pathway from the periphery. The electrical stimulation is applied to the median nerve at 0.6 Hz and recorded the EMG from the flexor and extensor muscles. (B) The 3D cortical rendering from the preoperative MRI. The location of the CS is shown with a red line (viewed here for patient 3). (P) Posterior, (A) anterior, (M) medial, (L) lateral. The electrode coregistration is based on the contralateral sensorimotor area on the craniotomy’s intraoperative image. The craniotomy image is to the right, with the white line representing the CS. (C) The recorded neural data with an ECoG grid from the contralateral sensorimotor area. The trace is annotated with the stimulation-induced spikes (S) in the bipolar EMG. (D) The overlapped ECoG SSEP trace and its derivative viewed 50 ms after stimulation onset, color-coded based on the presumed location (anterior-red, posterior-blue). The trace is superimposed with red marks representing the 1st N (negative) latency on the raw and derivative trace and 2nd P (positive) latency on the raw trace. (E) The spatial distribution of the SSEP at 1st N latency as an amplitude heat map on a 2D grid. The gray line represents the CS on the grid. (F) The unsupervised clustering of the SSEP trace. The clustering is applied to the second smallest eigenvector of the normalized Laplacian derived from the adjacency matrix (W). To the right, clustering results are visualized on the 2D grid by marking each contact with a color representing its membership. CS is represented with a gray line.

Patient demographics and clinical stimulation trials are listed in table 1. There were five men and three women with a mean age of 41.8 years. All the tumors involved the motor and/or sensory cortex. The subdural electrode grids were placed by the neurosurgeon, crossing the presumed location of the CS and hand knob, for each patient (eight in total), figure 1(B) (craniotomy). For patients 1–5, we used a 25–53 channel hybrid grid (CorTec GmbH, Frieburg Germany) with 10 mm spacing and 1–2.7 mm contact exposure. For patients 6–8, we used a 32–64 channel high-density grid with 5 mm spacing and 2.3 mm contact exposure (Ad-Tech, Michigan, MI). A 2 × 4 clinical grid (10 mm spacing), flipped, and placed under the dura, was used as the reference and ground. We recorded the neural data from the ECoG grids and bipolar surface electromyogram (EMG) from the forearm with a multichannel bio amplifier (gHIamp: 256 channels, g.tec medical engineering GmbH, Graz Austria) at a sampling frequency of 2.4 KHz with a 24bit A/D resolution. All behavioral and neural data were acquired, synchronized, and visualized in real-time intraoperatively using Simulink/Matlab and gHIsys block sets (g.tec medical engineering GmbH, Graz Austria).

### 2.3. Electrical stimulation

Two disposable conductive solid-gel electrodes were attached to the contralateral median nerve at the wrist. Using the clinical two or four channel EMG/EP Measuring System (Neuropack S1 MEB-9400), we stimulated with a frequency of 0.6 Hz (except P2 who received 4 Hz), a square wave electric pulse of 200 *μ*s, and a current intensity adjusted slightly between 5 and 15 mA. The stimulation caused small twitches of their thumb abductor pollicis brevis throughout the testing as recommended in the standard protocol (American Clinical Neurophysiology Society (ACNS), 2015) [41], figure 1(A). We used recorded bipolar EMG to capture the stimulation spikes. Over 100 stimulation trials were delivered for reproducible cortical responses to be identified. Subject information, and the number of recorded trials are given in table 1.

### 2.4. Co-registration of subdural electrodes

Anatomical landmarks such as hand knob, central sulcus, and blood vessels, viewed from photographs taken in the operating room, were used to determine the grid’s location on the brain. Incorporating a pipeline that was from our previous study [42], pre-op thin slice MRI scans of each patient was used to create a 3D cortical rendering of the brain from automatically segmented gray matter and white matter with SPM12 [43] and rendered in MATLAB (MathWorks, Natick, MA, USA). The CS and sensorimotor borders were retrospectively ascertained on the 3D rendering by two neurosurgeons, blinded to the electrophysiology. They did this according to the anatomical cortical landmarks like sulci, based on intraoperative navigation and tumor margins’ location to identify the sensorimotor gyri before SSEP monitoring. We coregistered the electrodes on the 3D cortical mesh, based on the craniotomy images with electrode placements, where we iteratively interpolated positions of contacts that were not visually exposed from the neighboring contacts figure 1(B). We used all channels anterior and posterior to the defined CS in our analysis and referred to them as the annotated regional channels.

### 2.5. The SSEP trace pre-processing and spatiotemporal visualization

We visualized and processed the ECoG data in MATLAB using our in-house developed toolbox with a graphical user interface (CNELAB 2017) [44]. We captured the stimulation spikes in surface EMG (figure 1(C)) and converted them to digital triggers to align the data for SSEP averaging. The SSEP trace was high-pass filtered at a cut-off frequency of 30 Hz as recommended by the ACNS (2015) [41], using the 2nd order Butterworth infinite impulse response filter sliding in forward and backward direction to prevent phase distortion (filtfilt function in MATLAB). We averaged the neural data over the stimulation trials after aligning the ECoG data to the stimulation onset trigger within a 40 ms post-stimulus window (10–50 ms). We then applied a Savitzky–Golay filter with a polynomial degree of 3 [45] to smooth the SSEP trace without distorting it and visualized the average trace in each channel to detect and exclude any corrupted channel. The corrupted channels were easily detected due to their large variance within the 40 ms window (see supplementary material, figure S1(A) (available online at stacks.iop.org/JNE/18/046038/mmedia)) and removed from the analysis. The most predominant succeeding components for the median nerve were the physiological postcentral negative to positive wave, N20-P30, described hare as the 1st negative (N) and 2nd positive (P), figure 1(D). We located the posterior 1st N latency between +17 and +25 ms, and the 2nd P latency between +27 and +40 ms. The 1st N or 2nd P amplitude was defined as the height between its lowest or highest peak and the preceding trough peak [35]. Intermediate peaks between +22 and +30 ms were superimpositions of the anterior P20 and the posterior P25 peaks and were not included in our statistical analysis. To inspect the temporal profile of SSEP, we produced overlay plots of the raw SSEP trace from the presumed anterior and posterior channels and marked the 1st N and 2nd P latency in the raw trace as red marks, figure 1(D).

After visual inspection, we noted that in some of the patients, the raw trace did not show a clear phase reversal. While the instantaneous amplitude of the SSEP was similar between channels located in M1 and S1, we noted that the change in the signal was not the same. Consequently, using an empirical approach, we computed the derivative of the SSEP within the same 10–50 ms post-stimulus window and investigated their information content separating M1 and S1 channels. Hence, we assessed the derivative trace in all our patients in comparison to the raw trace.

The amplitude distribution of the raw SSEP traces was visualized on a 2D plane (figure 1(E)) as a heat map using in-house designed visualization software (CNELAB 2017) [44].

We animated the spatiotemporal dynamics of the SSEP trace and its derivative on the individual 2D grid, with a smooth transition based on natural neighbor interpolation [46]. The 2D animation shows the SSEP peaks’ temporal alterations as heat maps, and at the preferred 1st N peak, we fused these 2D maps with the electrode grid position coregistered to the 3D rendering.

### 2.6. Assessment of discriminant time points in SSEP

To quantify the time points with the highest separability between anterior and posterior channels in the SSEP raw and derivative trace, we used the Fisher discriminative criterion (*F*), equation (1):

(1) $$F\left(t\right)=\frac{{\left(\mu_{A}\left(t\right)-\mu_{P}\left(t\right)\right)}^{2}}{\sigma_{A}^{2}\left(t\right)+\sigma_{P}^{2}\left(t\right)}.$$

where *μ*_A_ and *μ*_P_ are the mean of the anterior and posterior channels, and $\sigma_{A}^{2}$ and $\sigma_{P}^{2}$ are the respective variances in the channels for a particular time point (*t*) [47]. We visualized the *F*(*t*) for every sample point in the 10–50 ms post stimulus window and compared the most discriminative time points to the location of the 1st N and 2nd P peak in each subject. We further scrutinized the SSEP trace by computing the z-score normalization such that each channel had zero mean and unit variance and assessed the *F*(*t*) for these normalized traces as well.

### 2.7. Unsupervised classification with spectral clustering

After the delineation of the CS by neurosurgeons blinded to SSEP raw traces, the channels located anterior and posterior to the CS were referenced as our ground truth. We normalized the SSEP traces to retain its waveform morphology and then employed spectral clustering to group the data in an unsupervised fashion (figure 1(F)). Spectral clustering is an unsupervised machine learning technique that makes no assumptions on the shapes of the clusters [48, 49].

We used a Gaussian similarity function, equation (2), to create the adjacency matrix, *W*_*ij*_,

(2) $$W_{ij}=e^{-\frac{{\left|x_{i}-x_{j}\right|}^{2}}{2\sigma^{2}}}$$

which includes the Euclidean distance between the normalized SSEP traces, *x*_*i*_ and *x*_*j*_, of the channels *i* and *j*. The standard deviation, *σ*, was selected by running the algorithm repeatedly for different values of *σ* as suggested in [50]. We selected the value, *σ* = 2, which provided the least distorted clusters of the normalized trace, in all our patients. Here the entries of the adjacency matrix represent the connectivity network between different ECoG channels. We used the spectral graph theory to infer the data’s segmentation by applying *k*-means to the second smallest eigenvector of the normalized Laplacian matrix (*L*). The normalization of the graph Laplacian, derived from the adjacency matrix, was based on the random walk method (equation (3)) [45],

(3) $$L=I-D^{-1}W_{ij}$$

and *D*, the diagonal matrix, comprises the eigenvalues with elements:

(4) $$D_{ii}=\sum_{j}W_{ij}.$$

We used the eigengap heuristics [49] and the elbow point between eigenvalues (*D*) to choose the optimal number of clusters (*k*). Since spectral clustering does not require any supervisor input, the algorithm grouped the channels in an automated fashion based on the similarity between SSEP waveforms. Following the clustering, we color-coded each channel based on its membership. We visualized the color-coded clustering output over the electrode grid as depicted in figure 1(F).

In addition to clustering the channels of the entire ECoG grid, we conducted spectral clustering on multiple channel subsets of the large grid to simulate strip or small grids. To be more specific, the subsampled electrode combinations included 1 × *N* (strip) or 2 × *N* grid by taking the columns or the rows of the grid along the anterior-posterior direction based on the orientation of the electrode over the sensorimotor cortex.

### 2.8. Statistical analysis

We calculated the area under the receiver operating characteristic curve (AUC), to determine the accuracy in distinguishing the anterior channels from posterior channels and quantified the viability of delineating the CS. We calculated the AUC as the accuracy measure for the physiological SSEPs peaks and optimal Fisher time points. We used paired *t*-test to compare the quantified levels of separations, *F*(*t*), estimated from the raw trace and the derivative trace. We also compared the difference in the accuracy of physiological peak latencies 1st N and 2nd P. Finally, we evaluated the clustering performance of the raw SSEP and its derivative by estimating the clustering accuracy from the confusion matrix. More precisely, we determined the total number of correctly clustered anterior and posterior channels (CHL_CL_) based on the ground truth and calculated the accuracy defined in equation (5):

(5) $$\text{Accuracy}=\frac{100\times {\text{CHL}}_{\text{CL}}}{\text{Total}\;\text{number}\;\text{of}\;\text{channels}}.$$

## 3. Results

We recorded an average of 137 ± 73 stimulation trials from eight patients (P1–8). Two patients (P1 and P6) were fully awake, and the rest were sedated (see table 1) during the intraoperative recordings. Below, we present our findings on how the spatiotemporal attenuation of SSEP can be utilized to delineate the CS with heat maps and to cluster the anterior and posterior channels in an unsupervised fashion.

### 3.1. SSEP peaks

**Figure 2.**
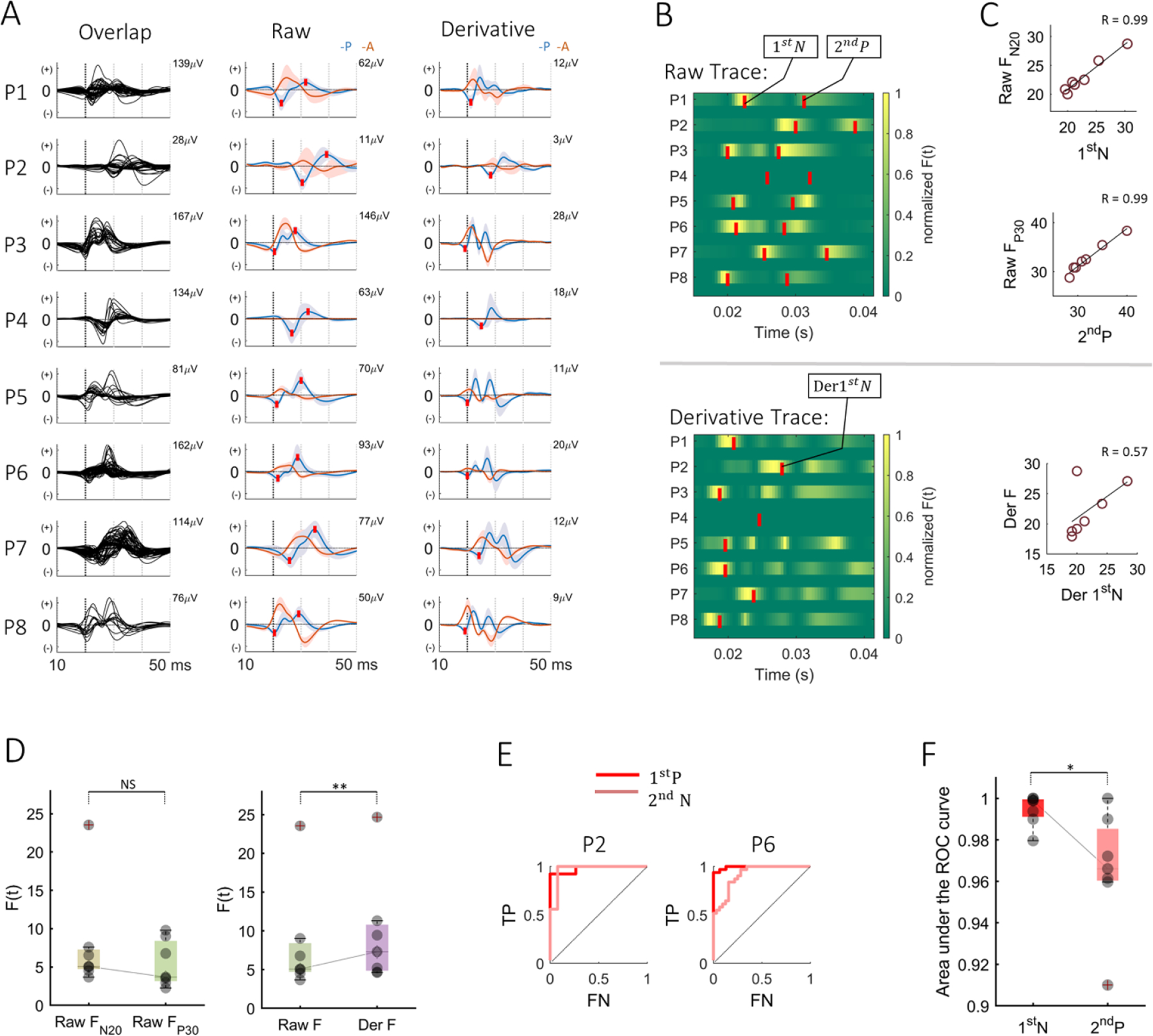
Peak and latency quantification: (A) the SSEP traces. The left insert shows the overlapped plot of the raw SSEP traces averaged over trials. Here, one cannot easily see the anterior and posterior regions. The middle and right insert shows the average raw and derivative SSEP traces for the anterior (A) and posterior (P) channels. The shaded region represents the variance. The 1st negative (N) and 2nd positive (P) peaks are shown as red marks. The 1st negative peak of the derivative trace (Der 1st N) is provided with a red mark to the right. (B) The temporal distribution of Fisher discrimination criterion, *F*(*t*), for all subjects. The yellow regions represent the maximum levels of separability between the anterior and posterior channels. The superimposed red marks on the images represent the 1st N and 2nd P latency of the raw SSEP trace (on top) and Der 1st N of the derivative trace (at the bottom). (C) The correlation between the Fisher discriminative time points and the physiological latencies. The top insert shows the correlation between 1st N peak latency and raw F_N20_. The middle insert shows the correlation between the 2nd P peak latency and raw F_P30_. The bottom insert shows the correlation between Der 1st N peak latency and the maximum derivative trace (Der F) (D) the Fisher level assessment. The left insert shows the box plot comparing the differences in the level of separation between Raw F_N20_ and Raw F_P30_. The right insert compares the difference between the maximum *F*(*t*) of the raw trace (Raw F) and Der F. (E) The ROC curves of P2 and P6. (F) The box plot compares the accuracy level at the physiological 1st N and 2nd P peak for all patients. It is based on the area under the ROC curves (AUC). Note: **p < 0.01, *p < 0.05, and NS: non-significant.

Figure 2(A) shows the overlapped plot of the raw SSEP traces and the average plot of the raw and derivative trace based on the annotated regional channels. The results obtained from normalized SSEP trace are provided in supplementary figures S1 and S2. The appraisal of the raw trace in each patient revealed the variability of the peak latencies with an average 1st N latency of 23.3 ± 3.6 ms (N20) and an average 2nd P latency of 31.8 ± 3.8 ms (P30). However, without prior knowledge of the anterior and posterior channels, it was difficult to localize these time points in figure 2(A) (left) and in supplementary figure S1(C). The average overlap of the raw SSEP traces of each patient was superimposed with the 1st N and 2nd P latency in figure 2(A) (middle) and in supplementary figure S1(D). Here, in some of our patients (P3 and P7), the instantaneous amplitude of the posterior channel 1st N was overlapped by the anterior channel which made it difficult to see a phase reversal.

In P4, the grid did not cross the CS and therefore did not have any anterior channels or a phase reversal in the SSEP trace. We also noticed that in three patients, (P3, P5, and P8), there existed a strong intermediate posterior positive peak, P25, between +20 and +22 ms. With the assessments of the derivative of the SSEP trace and the normalized derivative trace, a zero line was revealed between the presumed anterior and posterior channels from +17 ms to +22 ms in all our patients in figure 2(A) (right) and supplementary figure S1(G). We noted the well isolated 1st N peak of the derivative, which we termed as Der 1st N, and overlaid it on the trace. The isolated peak had an average latency of 22.1 ± 3.3 ms. We also noticed the multiple positive peaks which corresponded to the rate of change of the P25 and P30, however, along with the P25 peak of the raw SSEP trace, we did not further assess those peaks, as they were not consistent across subjects.

### 3.2. The most discriminative time points in SSEP trace

Figures 2(B) and S2(A) show the subject specific discriminative time points, *F(t)*, for the raw and the derivative SSEP trace, as a 2D image. The yellow patches represent the time points of maximum separation between the anterior and posterior channels. The analysis revealed two time points for the raw SSEP trace in figure 2(B) (top), in each patient, peaking at around 23.1 ± 3.1 ms and 32.7 ± 3.2 ms, which were termed as raw F_N20_ and raw F_P30_ respectively. We overlapped the relative 1st N (23.3 ± 3.6 ms) and 2nd P (31.8 ± 3.8 ms) latencies on the 2D image as red marks. We excluded P4 from the Fisher analysis since there were no anterior channels. Figure 2(C) (top and middle) and supplementary figure S2(B), showed that the prominent discriminative time points correlated with the 1st N peak latency (*R* = 0.987) and 2nd P peak latency (*R* = 0.986), where R represents the Pearson correlation coefficient. There was no significant difference in latency between the raw F_N20_ and 1st N latency (0.2 ± 0.8 ms, *p* = 0.2864) but a significant delay around 1.0 ± 1.0 ms (*p* = 0.0214) between raw F_P30_ and 2nd P latency. The raw F_N20_ and raw F_P30_, had relatively equal separation level, *F(t)*, (*F(t)* at F_N20_ = 7.92 ± 7.02; *F(t)* at F_P30_ = 5.58 ± 3.11, *p* = 0.1179) in figure 2(D) (left). The time points with the maximum level of separation in each of our patients were termed Raw F.

The time points with the maximum level of separation in each of our patients were termed Raw F. The quantification of separation based on the derivative trace, revealed scattered maximum discriminative time points (Der F), figure 2(B) (bottom). When overlapped with the first negative peak latency of the derivative SSEP trace, Der 1st N (22.1 ± 3.3 ms), these time points did not correlate (*R* = 0.568, *p* = 0.1834), figure 2(C) (bottom). When we compared the Raw F and Der F, there was a significant difference in the level of separation, figure 2(D) (right), where the maximum level of separation of the derivative trace was superior to the raw trace (*F(t)* at Raw F = 8.16 ± 7.02, *F(t)* at Der F = 9.55 ± 7.16, *p* < 0.01).

The ROC curves in figure 2(E) for the 1st N and 2nd P peak, revealed varying accuracy of separation. In some patients, as in P2 in figure 2(E) (right), the 1st N and 2nd P seemed indifferent. In another patient, as in P6 in figure 2(E) (left), the 1st N peak was more accurate. Across all patients, the overall assessment showed that the accuracy of separation of the 1st N (accuracy: 99.45 ± 0.76%) was significantly higher than the 2nd P (accuracy: 96.56 ± 2.88%) (*p* = 0.0144) in figure 2(F). When the SSEP trace was normalized, we noted that 1st N and 2nd P had similar accuracies (supplementary figure S2(C)). While the accuracy of 2nd P improved significantly with respect to unnormalized trace, the accuracy of 1st N was not different (supplementary figure S2(D)).

### 3.3. Spatial correlation with SSEPs peaks

**Figure 3.**
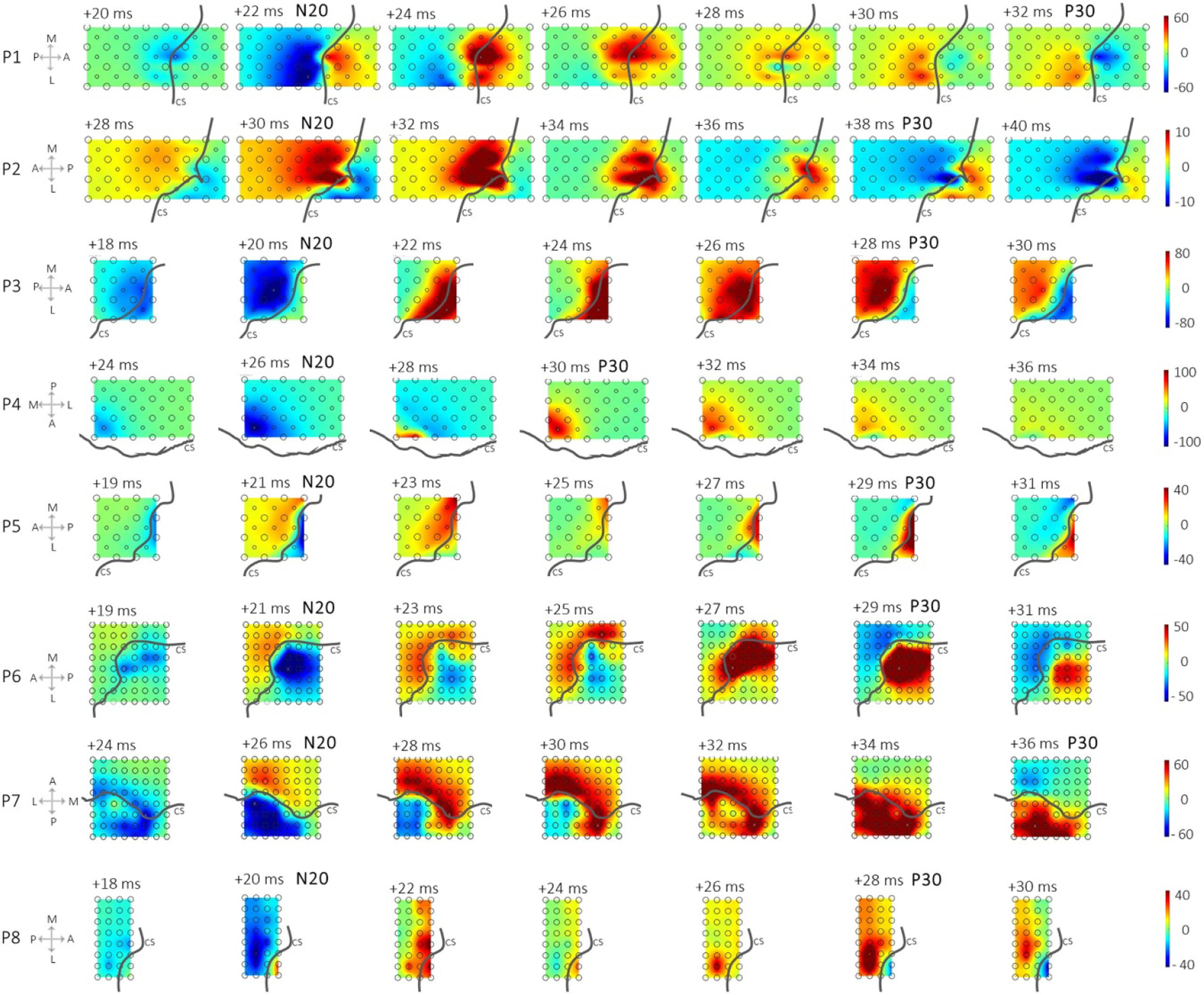
Temporal propagation of SSEP: the gray lines represent the CS. Each grid for each patient from left to right shows the temporal propagation of the SSEP from +18 ms, where the SSEP starts from channels in proximity to the CS, with a posterior propagation and a clear sensorimotor delineation at the physiological N20/1st N and then to an anterior propagation with a clear postcentral gyri definition at P30/2nd P ((P) Posterior, (A) anterior, (M) medial, (L) lateral, (CS) central sulcus). The maps are shown with a symmetric amplitude scale.

**Figure 4.**
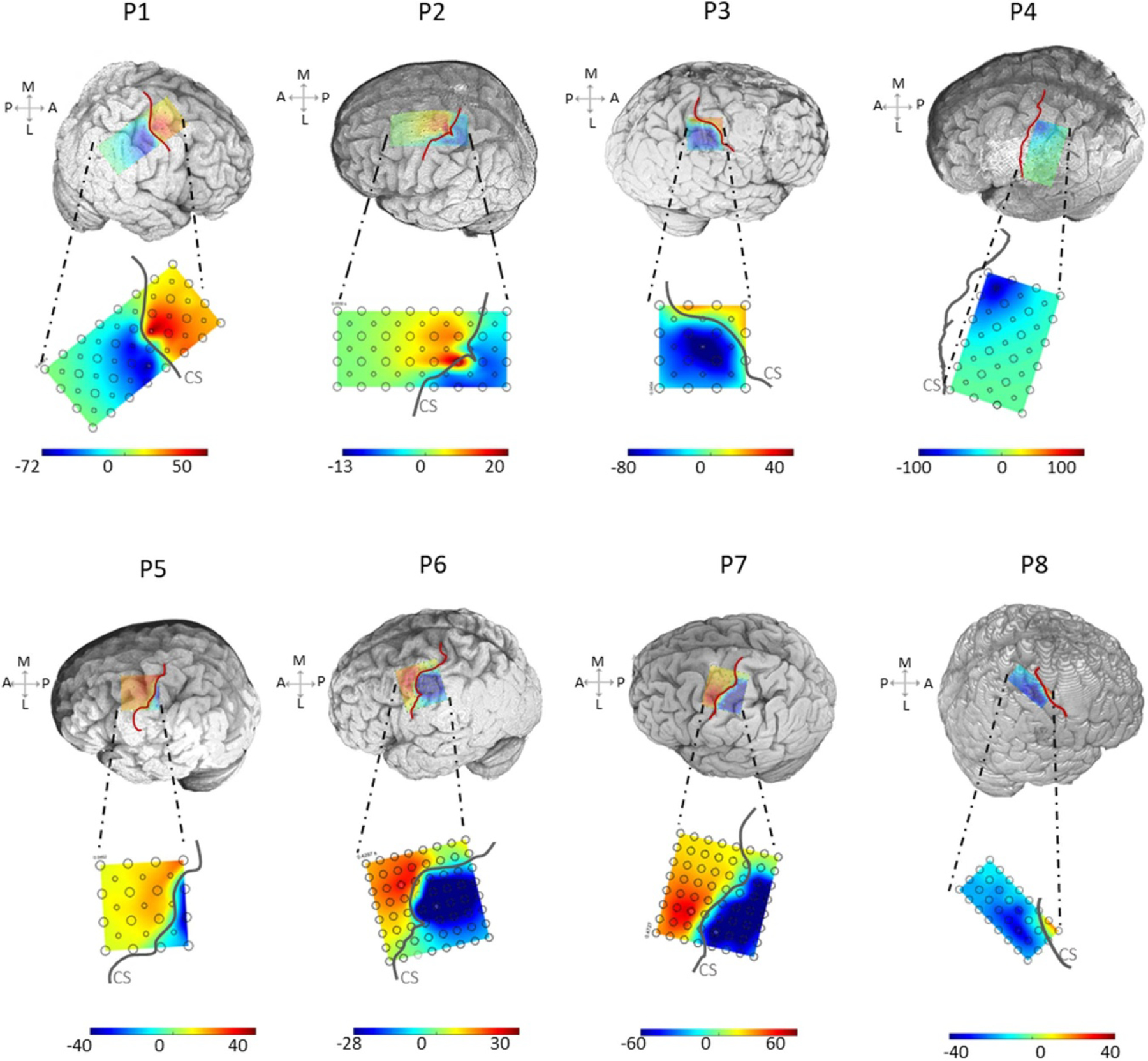
Asymmetric spatial heat maps on 3D rendering: spatial distribution of SSEP amplitude at the 1st N latency, shown on the 3D rendered brain and on the 2D grid for all eight patients. For each patient, the gray lines represent the CS on the 2D grid, and the red line represents the CS on the 3D brain. ((P) Posterior, (A) anterior, (M) medial, (L) lateral, (CS) central sulcus). In P4, the grid did not cross the CS. Yet, the gradient of the SSEP pointed towards the hand knob.

The spatial heat maps showed a contrasting color separation between the anterior and posterior regions in each patient in figures 3 and 4. The dynamic spatiotemporal evolution of the dipoles was best appreciated in the animated clips at 2 ms time intervals in figure 3. This showed the dipole initiation at the CS, with a posterior propagation and a clear postcentral gyri definition and CS delineation at the 1st N latency. The dipole then rotated displaying a clear postcentral gyri definition and once again vividly delineated the CS at the 2nd P latency. The asymmetric map in figure 4 at the 1st peak latency revealed a clear M1 and S1 definition on the cortical mesh. This spatial distribution in all our patients revealed the maximum deflection of the 1st and 2nd peak to be posterior-lateral to the hand knob. In P4, the grid did not cross the CS and there was no phase reversal. However, the gradient of the SSEP pointed towards the location of the hand knob.

### 3.4. Unsupervised spectral clustering

**Figure 5.**
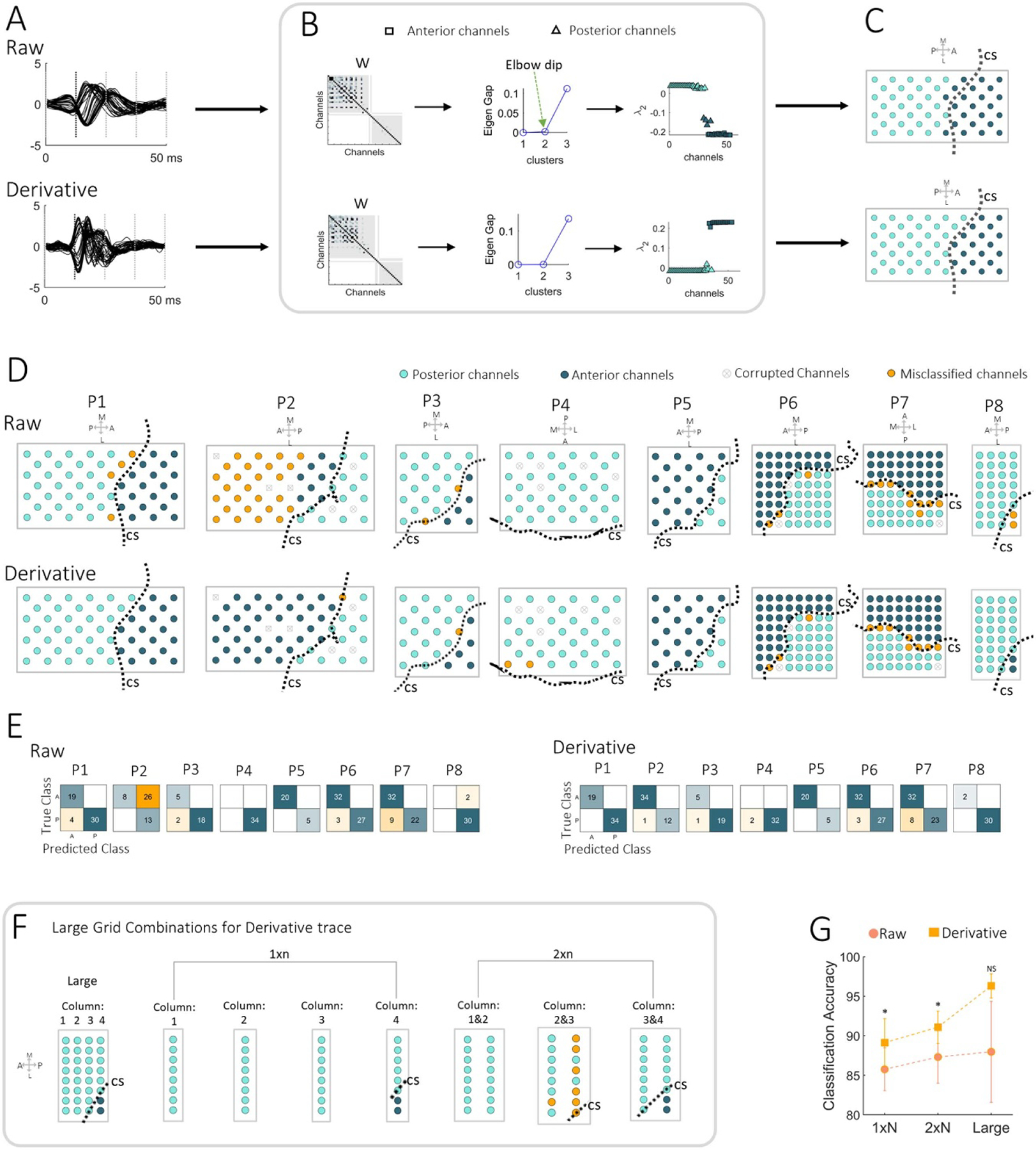
Spectral clustering analysis: (A) the overlay plot of normalized raw and derivative SSEP trace within the 10–50 ms window, viewed for P1. (B) The spectral clustering algorithm. The left insert shows the computed adjacency matrix used to derive the normalized Laplacian. The middle insert shows the eigengap heuristics. The elbow dip’s presence between three eigenvalues infers the number of clusters (*k*). The right insert shows the second smallest eigenvector, color-coded in shades of blue based on the output of *k*-means clustering (*k* = 2). (C) The clustering outcome marked on the 2D grid. The dotted line on the 2D grid is the CS line which represents the ground of truth. ((P) Posterior, (A) anterior, (M) medial, (L) lateral, (CS) central sulcus). (D) The spectral clustering of anterior and posterior channels in all our patients. The clustering results on the 2D grid are shown for the raw and derivative trace. The misclassified channels are reassigned to a different color (orange). The corrupted channels, not included in the clustering analysis, are shown with a gray cross. (E) The confusion matrix (A: anterior, P: posterior) shows the number of correctly clustered channels in the blue cells and the number of misclassified channels in the orange cells. (F) An illustration of the combinations of resampled grid of P8. Clustering results were obtained from the derivative trace. (G) The clustering accuracy compared between the raw trace and the derivative trace of the 1 × N, 2 × N, and the large grid. Note: **p < 0.01, *p < 0.05, and NS: non-significant.

Figure 5(A) shows the normalized raw and derivative SSEP traces of P1 used in the spectral clustering. SSEP traces of all subjects are provided in supplementary figure S1. As demonstrated in figure 5(B), we estimated the ultimate number of clusters from the presence of the elbow-dip between the smallest sorted eigenvalues of the normalized Laplacian. The optimal number of clusters (*k*) was 2 for the large grid of P1 as shown in figure 5(B) (middle). We also noted this cluster assignment (*k* = 2) in most of our patients, and in the majority of the resampled grids. The *k*-means clustering applied to the second smallest eigenvector provided a color-coded classification as shown in figure 5(B) (right), which we projected onto the 2D grid in figure 5(C). Based on the clustering of the raw and derivative SSEP trace, we marked the anterior and posterior channels with two different colors on the 2D electrode grid as shown in figure 5(D). However, there were a few instances where only one cluster was suggested such as the raw trace of P4 and P8.

The confusion matrices are provided in figure 5(E) and the accuracies for the various electrode sizes are shown in figure 5(G). We saw that the clustering based on the derivative trace had fewer misclassified channels (orange cells) than the raw trace. Even though 2/8 of our patients (P5 and P6) had equal accuracy for the raw and derivate, the accuracy of the derivative SSEP trace on the large grid (accuracy: 96.3 ± 4.3%) was higher than the raw SSEP trace (accuracy: 88.0 ± 18.1%) (*p* = 0.1227).

Figure 5(F) demonstrates the nature of resampled grid for P8 and associated clusters. Due to the lack of a clear elbow in the eigen spectrum, a single cluster was used in several combinations of the large grid (see also supplementary figure S4). As we moved up in electrode size from the 1 × N strip to the large grid, the clustering accuracy increased systematically for the raw and derivative trace as shown in figure 5(G). Table 2 summarizes the classification accuracy for each type of ECoG grid.

## 4. Discussion

The main objective of the present study is to verify that the CS can be delineated with spatial heat maps assessed with ECoG grids, and the sensory and motor cortices can be determined without manual supervision in the presence of high number channels. This is important due to the need to locate the eloquent cortex in a short time without electrode replacement or relocation. Our results show that heat maps vividly delineated the CS at the physiological N20/1st N and P30/2nd P. The automated unsupervised clustering provided a clear anterior and posterior channel separation without the need for peak and latency tracking in the presence of high numbers of recording channels.

### 4.1. Spatiotemporal evolution and interpretation of SSEPs

SSEP is generated from electrical stimulation to the median nerves at the periphery, where the signal is transmitted via the dorsal root ganglion to the medial lemniscus. The signal then propagates to the thalamus’s contralateral ventral posterolateral nucleus before arriving at the primary somatosensory cortex for processing, in figures 1(A) and (B). Previous works have shown that the N20 reflects the excitatory postsynaptic potentials of the afferent thalamocortical volley [28]. It has also been repeatedly reported that the N20 and the P30 are generated from the posterior wall of the CS Brodmann area (BA) 3b, while the intermediate P25 is from BA 1 [30, 35, 51, 52].

The ANCS (2015) [41] suggested a minimum of 16 electrodes for intraoperative monitoring. However, clinical investigation of the CS utilizes strip electrodes. In practice, the recording strip electrodes are commonly placed at a close location to the generator site, while a ‘reference’ electrode is placed as far away as possible [28]. However, when the interelectrode distance becomes too large, it is easily influenced by electrocardiogram and muscle artifacts, which increases the complexity of the ECoG analysis [4, 25, 28]. These difficulties lead to multiple strip placements on the craniotomy [21, 24, 25, 32]. Furthermore, the routine clinical MSSEP-PRT relies only on the amplitude construal of the conventional N20 (posterior) and P20 (anterior) between the two adjacent contacts to delineate the CS. In some cases, the typical phase reversal with the strip at the 20 ms is ambiguous [33]. This may be due to cortical displacements, caused by either the tumor invasion or peritumoral edema [4]. It may also be due to the placement of strip electrode away from the localized nature of the hand area [24, 33]. Several investigators have described how several recordings with the use of a single strip in different locations might be needed to find the appropriate position to target the somatosensory cortex [13, 25, 29, 31, 33, 53], whose success needs to be further corroborated by DCS [4, 24, 35] when there is persistent doubt. The multiple electrode relocation and placements require significant time and the additional risk of hemorrhage and trauma, while DCS requires a rigorous anesthetic regimen [39] and may induce seizures that could jeopardize the surgical procedure or complicate the motor monitorization. With a single placement of the ECoG grid, we can identify the different parts of the craniotomy with the animated heat map to target the active areas with ease and use the spatial orientation to assess the anterior and posterior regions and delineate the CS.

### 4.2. Correlations of hand area to the spatial heat maps

The technique of viewing the spatial distribution of SSEPs N20 as a heat map to delineate the CS with a grid was recommended by numerous studies decades ago [14, 29, 35, 54, 55]. In this study, the temporal propagation of the SSEP was viewed as a symmetric spatial amplitude map for each patient in figure 3. It revealed the clear delineation of the CS at the two different time points corresponding to the N20 and P30. A transcranial magnetic stimulation study showed that current flow across the CS is from, a posterior, to an anterior direction [56]. It supports our findings that the dipole formation starts from the CS and posterior channels near the CS, where it dominates over the hand area, revealing the maximum N20. The dipole then rotates from the posterior region to the anterior region, yielding the negativity to propagate to the anterior direction. The peaks from the spatial heat map in figures 3 and 4 were diffused throughout the grid with an elevated gradient in proximity to the CS. The map revealed the corresponding motor area when viewed asymmetrically in figure 4. We have also shown that most physiological activities do not occur posterior-medial to the hand area but showcase a posterior-lateral orientation. Wood *et al* explained that the medial postcentral locations show little to no N20 due to the superposition of the early portion of P25 [29]. They also emphasized that immediate negativity is a useful localization criterion of a postcentral recording, though an initial positivity does not necessarily indicate a pre-central recording [29]. Based on that study, we were motivated to prioritize the first negative peak for the raw and derivative trace, rather than the positive peaks. Previous works have shown that the primary sensory hand area coincided with the maximum N20 deflection [52]. We too noted that, in most of our patients, the sensory hand area coincided with the largest peaks at the N20 and P30 (also the raw F_N20_, and the raw F_P30_ in supplementary figure S3). These peaks exhibited a lateral orientation to the hand knob in figure 4, which was first seen by Wood *et al* [29], for the 1st N/N20 peak. Wood *et al* showed that due to the bend on of the CS in the hand area, which commonly forms a convex cap of tissue pointing toward the frontal midline, there exists an ‘on-axis’ line passing through the maxima of the N20 and P30, forming an acute 70° line nearing the overall course of the CS [13, 24, 29].

Using a high-density ECoG grid to visualize the heat map, we were able to delineate the CS vividly with a high spatial resolution. The ECoG grid makes it possible to sample a larger surface area over the sensorimotor gyri. Therefore, the gradient of the SSEP can be seen in all directions, with the CS line drawn out. However, assessing the SSEP over a line with a strip electrode limits the view of the sensorimotor area as a heat map and may not clearly illustrate the CS delineation. Additionally, in cases where the grid does not cross the CS (such as P4), the gradient of SSEP helps to localize the hand knob or the direction of the source, which in turn can guide the electrode replacement.

### 4.3. The quantified time points of discrimination

The use of the N20–P30 to define the postcentral gyrus and the N20 to delineate the CS has been established in previous studies [14, 19–22, 35, 57–59]. In contrast, using P30 peak to determine the CS can be challenging due to the overlap of the small primary negativity of BA1 [35], seen by its relatively low accuracy in figure 2(F). The visual assessment of the normalized SSEP trace did not improve the peak latency interpretation for patients without prior or proper phase reversal in supplementary figures S1(C) and (D). While the accuracy at the conventional 1st N compared to the un-normalized trace did not improve (supplementary figure S2(D)), the normalization improved the accuracy at the 2nd P significantly (supplementary figure S2(E)). We anticipate that normalization in the routine clinical assessment of the SSEP trace might contribute to the peak latency interpretation if there is an apparent phase reversal.

Allison *et al*’s model of electrogenesis based on electric field theory showed that the calculated equal distribution due to the BA 3b primary positivity and negativity existed at two points in time (18 ms and 30 ms) [35]. Their results coincide with our Fisher estimation of the two unique time points within the 40 ms window of the raw SSEP trace, one close to N20 and the other slightly later than P30. Based on our quantified time points of separations in figure 2(B), the two different time points, raw F_N20_, and raw F_P30_, had the highest level of separation between anterior and posterior channels in figure 2(D) (left), and heavily correlated with the physiological 1st N and 2nd P latencies in figure 2(C) (top and middle).

Due to the nature of overlap of the instantaneous amplitude of anterior and posterior channels, as seen in P3 and P7, the assessment of the derivative revealed a zero line that separated the presumed sensory and motor channels within the first 20 ms. We believe it to be due to the dipole formation since, before that point, there is a uniform intersection between the anterior and posterior channels in figure 2(A) (right). Although the derivative SSEP showed scattered discriminative time points that did not correlate with the Der 1st N in figure 2(B) (bottom), and figure 2(C) (bottom), the maximum discrimination of the derivative was superior as shown in figure 2(D) (right). Although there are unique time points, two in the raw trace and one in the derivative trace, exist within the first 50 ms of the SSEPs, these time points need be tracked to delineate the CS.

### 4.4. The unsupervised delineation of central sulcus

Manual selection of peak latency is difficult and relies profoundly on expert interpretation. The large number of channels of the ECoG grid makes visual inspection difficult in figure 2(A). With the need to differentiate between the anterior and posterior channels in a short amount of time, unsupervised spectral clustering was employed. Spectral clustering uses subspace decomposition on high dimensionality to achieve data clustering. Some studies adopted spectral clustering to identify and characterize the connectivity between cortical areas based on fluctuations in high gamma power [60]. Others have employed it to analyze the grouping of the channels at different stages of seizure, based on their average mutual interactions [61]. Spectral clustering has also been used to help determine epileptic focus by extracting features that cluster different regions of the brain based on their functional dependencies [62].

In this study, using both the raw and derivative trace, spectral clustering managed to separate the anterior and posterior channels without peak interpretation or expert supervision. The approach was based on the morphology of SSEPs originating from the S1 and M1. We noted that the derivative provided a higher accuracy than the raw SSEPs. We believe this to be due to its higher discriminative power estimated from the Fisher analysis in figure 2(D) (right).

We studied the influence of the electrode size on clustering accuracy by resampling the channels of the large grid to form 1 × N or 2 × N electrodes. There was a systematic increase in accuracy from 1 × N to 2 × N to a large grid, for both the raw and derivative trace. Assessing a larger number of channels also enabled a better, or more accurate, estimation of the connectivity matrix (and the Laplacian) that depended on the waveform morphology of different cortical areas. In contrast, the sampled 1 × N strip showed a relatively lower accuracy. We hypothesized this to be due to the sampled 1 × N electrode being away from the primary SSEP source in some cases and lacking an apparent phase reversal. We saw this in multiple instances, from the rows of P3 and P5 to the columns of P4, P6, and P8, in figures 5(D), 5(F), and S4. While we seek two clusters in ideal situations, single clusters based on lack of elbow dip confirmed the improper CS crossing or lack of a clear phase reversal. Therefore, we believe that our clustering method applied with a small grid, or strip electrode, will be an extra precaution when the grid has not crossed the CS or poorly placed.

## 5. Conclusions

Relying on the phase reversal around N20 in SSEPs continues to be an undisputed gold standard that is still very accurate in the mapping of the eloquent cortex. However, in those grid placements where only a portion of the grid is exposed or the electrode is slightly away from the SSEP source, the phase reversals are challenging to interpret. We observed that the spatial distribution of the SSEPs visualized as a spatial heat map could delineate the CS and define the primary sensorimotor areas at various time points. In particular, the unsupervised clustering may help to confirm the CS delineation further while using the entire derivative SSEP trace from 10 to 50 ms, without the need to track the N20 or any other peak latency. Intraoperatively, cortical mapping requires online processing of the neural data, and our proposed approach can be easily executed in real-time due to its low computational complexity. The spatial heat map and clustering may overcome the limitations of peak interpretations, and the need for multiple strip placement or relocations due to the cortical displacement or tumor invasion. The approach can improve the CS’s delineation for the localization of the sensorimotor region.

## Supplementary Material

Suppl Material

## Figures and Tables

**Table 1.** Patient demographic data and stimulation trials.

Patient ID	Hemisphere	Tumor infiltration area	Trials	State
P1	Right	Motor and sensory	66	Sedated
P2	Right	Motor and premotor	281	Awake
P3	Left	Motor and premotor	164	Sedated
P4	Left	Motor and premotor	80	Sedated
P5	Left	Motor and sensory	95	Sedated
P6	Left	Sensory and parietal lobule	200	Awake
P7	Left	Motor and sensory	101	Sedated
P8	Right	Frontal lobe and premotor	105	Sedated

**Table 2.** Accuracies obtained with different electrode sizes.

	Raw	Derivative
Grid type	Accuracy %	
1 × N	85.8 ± 7.7	89.1 ± 8.6^a^
2 × N	87.3 ± 9.4	**91.1 ± 5.8** ^a^
Large	88.0 ± 18.1	**96.3 ± 4.3**

a Represents significant difference between each column (*p* < 0.05). Bold font represents significant difference between each row (*p* < 0.05).

## Data Availability

The data generated and/or analyzed during the current study are not publicly available for legal/ethical reasons but are available from the corresponding author on reasonable request.
