# Supplementary material for: Unsupervised machine learning can delineate central sulcus by using the spatiotemporal characteristic of somatosensory evoked potentials
Source: J Neural Eng. Author manuscript; Available in PMC 2021 Dec 30. (PMC8718352; doi:10.1088/1741-2552/abf68a)
Supplement: Suppl Material [file NIHMS1766028-supplement-Suppl_Material.pdf]

SUPPLEMENTARY MATERIAL

1. The progressive visual assessment of the normalized SSEP trace

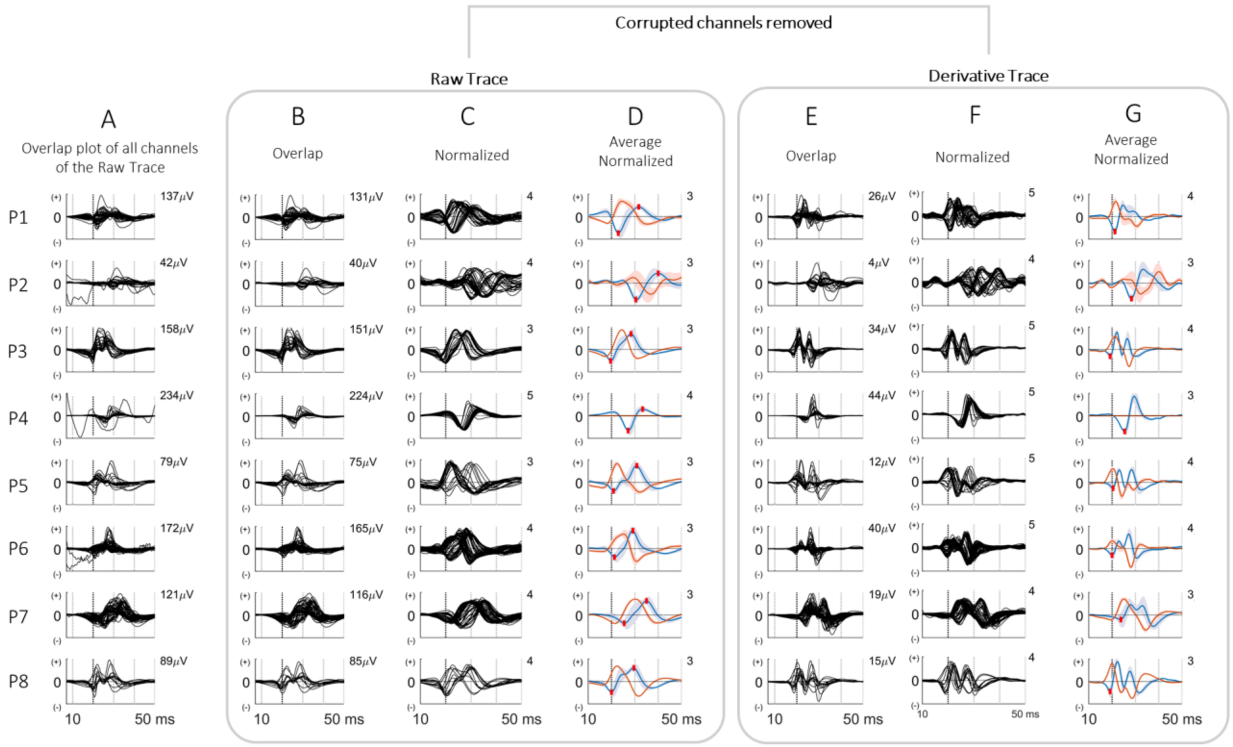

Figure S1: SSEP trace assessment: (A) The overlay plot of raw SSEP trace from all channels without excluding the corrupted ones. **(B)** The overlap plot of the raw SSEP trace after removing the corrupted channels. **(C)** The overlap plot of the raw SSEP trace after normalization. **(D)** The average plot of the normalized raw trace based on the presumed anterior (A) and posterior (P) channels. The shaded region represents the variances of each channel. The normalized (n) 1^{st}N and (n) 2nd P peaks is shown as red marks on the raw trace. **(E)** The overlap plot of the derivative SSEP trace. **(F)** The overlay plot of normalized derivative SSEP trace. **(G)** The average plot of the normalized derivative trace based on the presumed anterior and posterior channels, with the shaded variances and 1st N peak of the derivative trace (nDer 1st N) overplayed as red marks.

2. The most discriminative time points in the normalized SSEP trace

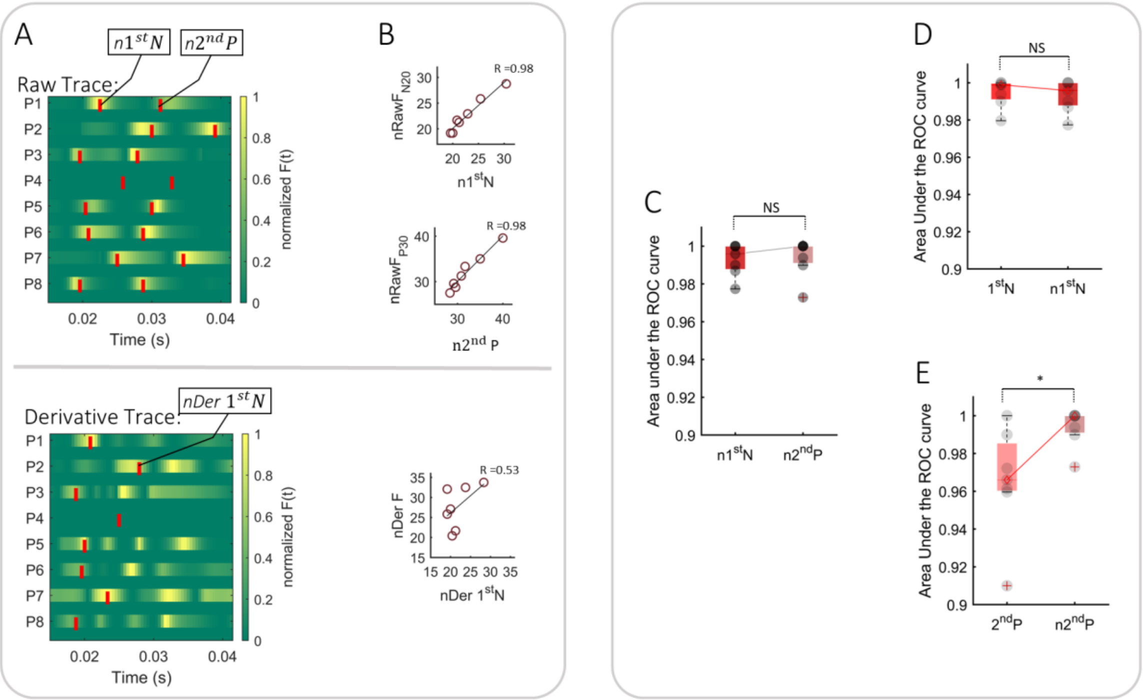

Figure S2: Peak and latency quantification of normalized trace: **(A)** The temporal distribution of the Fisher discriminative criterion, F(t), for the normalized trace for all subjects. The yellow regions represent the maximum levels of separability between the anterior and posterior channels. At the top, the red marks represent the (n) 1st N and (n) 2nd P latency. At the bottom, the red marks represent the (n)Der 1st N latency. **(B)** The correlation between the most discriminative time points (y-axis) and the physiological time points (x-axis) estimated from the normalized trace. The top insert shows the correlation between the (n)Raw F_{N20} and the n1st N peak latency. The middle insert shows the correlation between the nRaw F_{P30} and the n2nd P peak latency. The bottom insert shows the correlation between the maximum nDer F and the nDer 1st N peak latency ($p = 0.2179$). **(C)** The accuracy levels based on the area under the ROC curves (AUC) for all patients normalized raw SSEP trace at the physiological peaks, compared between the n1st N (99.3±0.86%), and the n2nd P (99.4±1.01%). **(D)** The accuracy level compared between the un-normalized raw trace at the 1st N and normalized raw trace at the n1st N. **(E)** The accuracy level compared between the un-normalized raw trace at the 2nd P and normalized raw trace at the n2nd P (right). The n2ndP is more significant than the 2ndP. Note: **p<0.01, *p<0.05, and NS: non-significant

3. The spatial correlation between physiological time points and Fisher discriminatory time points

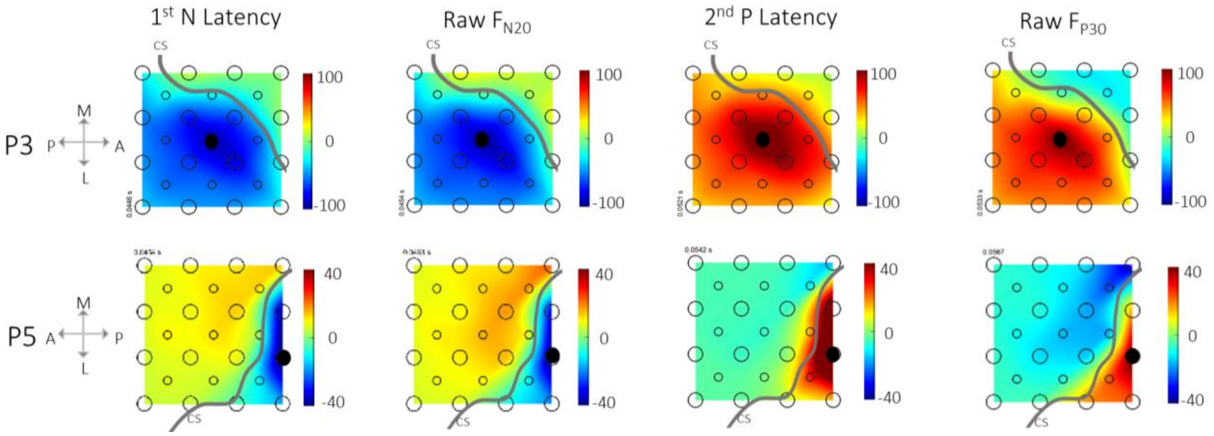

Figure S3: The symmetric spatial heat maps, shown here for P3 and P5, at the 1st N latency, raw F_{N20}, the 2nd P latency, and the raw F_{P30}. There is equal separation and clear delineation of the CS at the at these time points. The channel with the maximum peak latency is shown as the black dot on the grid, and the gray lines represent the CS. ((P) Posterior, (A) Anterior, (M) Medial, (L) Lateral, (CS) Central Sulcus)

4. An illustration of unsupervised clustering applied to P4:

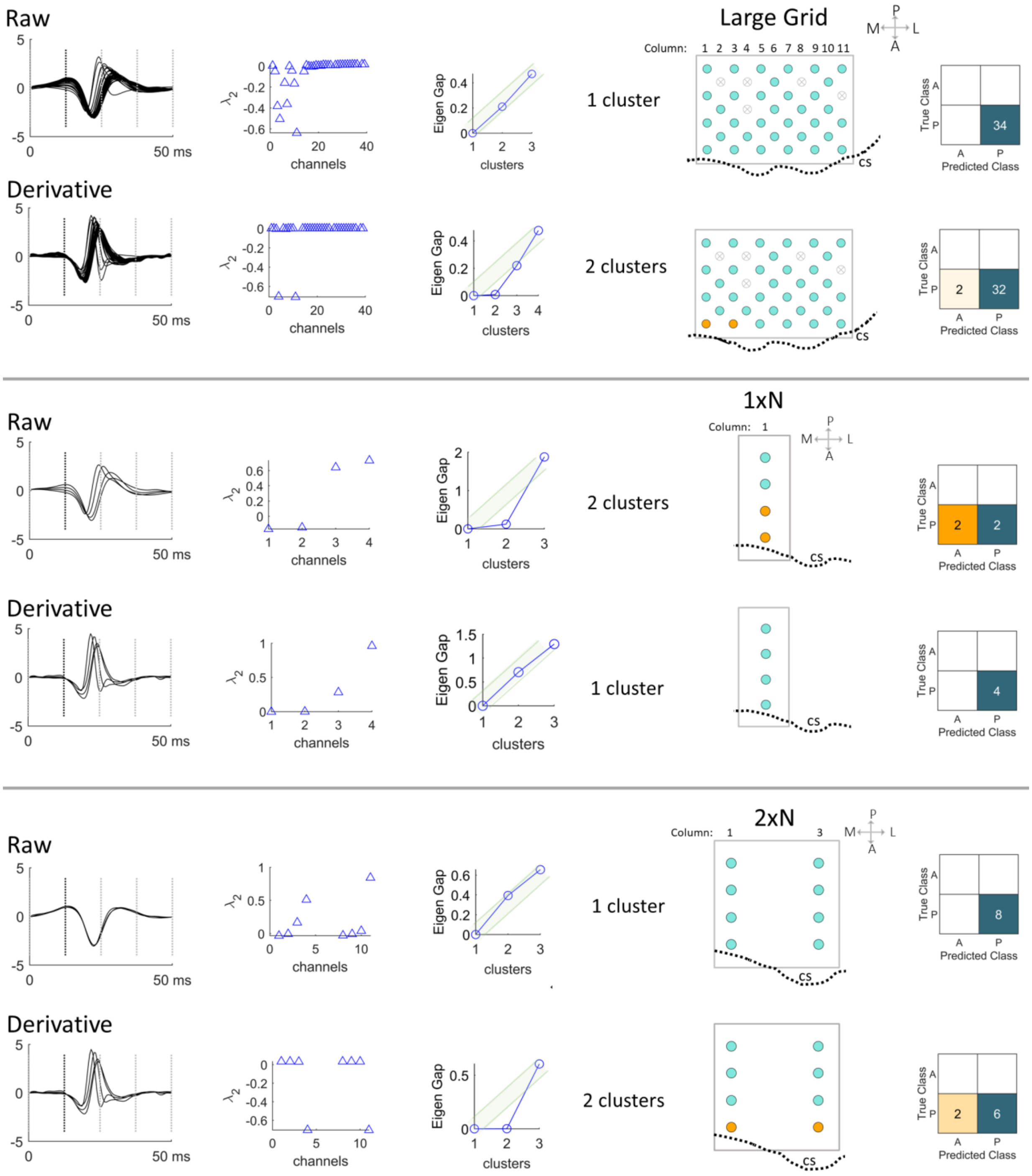

Figure S4: Spectral Clustering on several combinations of the large grid, for P4: The normalized SSEP trace is shown in the first column in each section. The second column shows the second smallest eigenvector of the normalized Laplacian. In the third column, the gap heuristics to determine the optimal number of clusters. The fourth column shows the clustering results projected on the 2D grid, where the CS is marked with a dotted black line. The associated confusion matrix is shown in the fifth column. **(Top)** The spectral clustering results obtained with the large grid. The gap heuristics infer 1 cluster for the raw trace and 2 clusters for the derivative trace. The misclassified channels were marked with orange color. **(Middle)** The spectral clustering results obtained from 1xN electrode (the first column of the large grid to simulate a 1xN strip electrode). It fails for the raw trace with two misclassified channels. (**Bottom)** The spectral clustering applied to column 1 and column 3 of the large grid to simulate a 2xN grid. Two channels were misclassified and marked with orange.
